# Bidirectional Transfer between Metaphorical Related Domains in Implicit Learning of Form-Meaning Connections

**DOI:** 10.1371/journal.pone.0068100

**Published:** 2013-07-03

**Authors:** Xiuyan Guo, Fengying Li, Zhiliang Yang, Zoltan Dienes

**Affiliations:** 1 Shanghai Key Laboratory of Magnetic Resonance and School of Psychology and Cognitive Science, East China Normal University, Shanghai, China; 2 Department of Psychology, Zhejiang Normal University, Jinhua, China; 3 School of Psychology and Cognitive Science, East China Normal University, Shanghai, China; 4 Sackler Centre for Consciousness Science and School of Psychology, University of Sussex, Brighton, United Kingdom; Emory University, United States of America

## Abstract

People can implicitly learn a connection between linguistic forms and meanings, for example between specific determiners (e.g. this, that…) and the type of nouns to which they apply. Li et al (2013) recently found that transfer of form-meaning connections from a concrete domain (height) to an abstract domain (power) was achieved in a metaphor-consistent way without awareness, showing that unconscious knowledge can be abstract and flexibly deployed. The current study aims to determine whether people transfer knowledge of form-meaning connections not only from a concrete domain to an abstract one, but also vice versa, consistent with metaphor representation being bi-directional. With a similar paradigm as used by Li et al, participants learnt form- meaning connections of different domains (concrete vs. abstract) and then were tested on two kinds of generalizations (same and different domain generalization). As predicted, transfer of form-meaning connections occurred bidirectionally when structural knowledge was unconscious. Moreover, the present study also revealed that more transfer occurred between metaphorically related domains when judgment knowledge was conscious (intuition) rather than unconscious (guess). Conscious and unconscious judgment knowledge may have different functional properties.

## Introduction

Humans are equipped with powerful learning mechanisms for acquiring knowledge of regularities in the environment without awareness [1]. This form of learning, known as implicit learning, is manifested in language acquisition. There is abundant evidence that people can implicitly acquire syntax of languages, including some properties of finite-state grammars (e.g., [1]–[4]), symmetries or recursive embeddings (e.g., [5]–[7], and word order (e.g., [8]). Implicit learning also contributes to vocabulary acquisition, especially to the acquisition of form-meaning connections [9]–[13], which lie at the heart of language processing and learning [14], [15]. This paper will explore the nature of the implicit acquisition of form-meaning connections.

Williams' work [13] provides strong evidence of implicit learning of form-meaning connections. In one study (Experiment 1), participants first had to learn four novel words (gi, ro, ul and ne, which were introduced as determiners), which encoded an explicit meaning dimension (gi and ro occurred with near objects, ul and ne with far objects) and a hidden meaning dimension (gi and ul were used with animate nouns and ro and ne with inanimate nouns). The novel words were embedded in English carrier phrases or sentences, as in “At the fair they threw balls at ne plates”. After training, participants were tested on the animacy rule (the hidden meaning dimension) in a generalization task. The results showed that though participants reported no awareness of the relevance of animacy, they performed significantly above chance. These results suggest that people can unconsciously acquire form-meaning connections.

Form-meaning connections can have a complex structure. One form may encode multiple meanings at once [16], such as literal and metaphorical meanings. For example, “big” means “of considerable size” literally (e.g., a big plaza) and “having or exercising considerable authority” metaphorically (e.g., a big official). Importantly, these two kinds of meanings are closely correlated with each other. Activation of the meanings of height or size primes the meaning of power automatically [17], [18]. Given the close association between the meanings of metaphorically related domains, it is plausible that as long as people acquire form-literal meaning connections of a concrete domain (e.g., height or size), they can automatically establish form-metaphorical meaning connections of an abstract domain (e.g., power), producing a metaphor-consistent transfer.

Li et al. [19] tested this hypothesis by adopting Williams' paradigm [13]. Participants learnt form-meaning connections of a concrete domain (height) and were tested on two types of generalization (literal generalization: height vs. metaphorical generalization: power). The results not only replicated, but also extended the findings of [13], by demonstrating that participants unconsciously established form-meaning connections of a concrete domain (but for height rather than animacy) as well as that of an abstract one (power) in a metaphor-consistent way, suggesting transfer of knowledge from a concrete domain to an abstract one was achieved. Li et al's study [19] provided strong evidence in support of Reber's argument [2] that implicit knowledge can flexibly apply to new domains (see also [3], [4], [20], [21]). If implicit knowledge is flexible, then it may allow transfer of form-meaning connections in the opposite direction (i.e., from abstract to concrete).

This question is also motivated by theories of the directionality of metaphor. Lakoff & Jonson [22], [23] argued that a hallmark of metaphor is its cognitive asymmetry or directionality, implying that knowledge or experience of a concrete domain should affect that of an abstract one, but not vice versa. Supporting evidence comes from research demonstrating that spatial displacement affected estimates of duration, but duration did not affect estimates of spatial displacement [24]. In contrast, evidence has also shown the reverse effect of an abstract domain on a concrete one (e.g., [25]–[27], suggesting that psychological consequences of metaphors can be bidirectional. The reverse causal effect of an abstract domain on a more concrete one can be easily handled by grounded cognition theories (e.g., [28], [29]), which posit that abstract concepts are typically grounded in concrete experience, such as simulations, situated action, and bodily states. According to this perspective, abstract and concrete concepts are closely intertwined and their activation co-occurs [30]. Thus, there is no need to postulate an asymmetrical influence between metaphorically related domains [31]. Processing either concrete or abstract information may activate its metaphorically associated information, resulting in bidirectional influences between both domains.

One major goal of the current study was to explore the bidirectional transfer of knowledge of form-meaning connections between a concrete domain and an abstract one. In particular, grounded cognition approaches predict that people should be able to unconsciously transfer knowledge of form-meaning connections from a concrete domain to an abstract one, and vice versa. We tested this conjecture by having participants learn form- meaning connections of different domains (concrete vs. abstract). Their subsequent performance on two kinds of generalizations (same and different domain generalization) served as the dependent variable. If influence is bi-directional, people who are trained on different domains should successfully transfer the acquired knowledge from either training domain to the corresponding generalization domain. As size is also a key dimension of spatial mappings of ranks [32], which is strongly associated with power [18], the current study sought to investigate the bidirectional effect by using the size representation of power, namely, big = powerful, small = powerless [18]. In contrast with the relative size distinction used in previous studies [9], [11], which found no learning, the current study used a natural size distinction, which, we will show, is an intrinsic feature of an object. Intrinsic features may be automatically encoded and, thus, facilitate implicit learning.

Another issue of interest in the present study was the conscious status of the knowledge. Central to the question of whether knowledge transfer occurs unconsciously is whether the judgment knowledge itself is conscious, or structural knowledge underlying the judgment is conscious [4]. According to Dienes and Scott [33], judgment knowledge is knowledge about whether a particular test item has the same structure as the training items, while structural knowledge is knowledge of the structure of the training items that allows that judgment. For example, knowing the form-meaning association is structural knowledge; knowing that a particular sentence is grammatical is judgment knowledge. Both structural knowledge and judgment knowledge can be conscious or unconscious. Based on the distinction between judgment and structural knowledge, Dienes and Scott [33] developed trial-by-trial structural knowledge attributions to precisely assess the conscious status of the knowledge (see also [9], [34] for additional applications of these measures to language learning). Specifically, on each trial participants are asked to state the basis of their judgment: Was there no basis at all, they were purely guessing; did it have a basis but they have no idea what it was, they just used intuition; or did it have a basis, and they knew the basis, it was either a rule they could state or else they recollected seeing a similar example before. Guess and intuition correspond to cases where the knowledge of the underlying structure, i.e. of the rule, is unconscious in that the participant claims not to know the structure. In these cases, structural knowledge is unconscious. Rules and memory correspond to conscious structural knowledge in that the participant claims to know either a rule, or a previous example that serves as an analogy. From our previous research using power [19], we expect participants to almost entirely use the unconscious structural knowledge attributions, guess and intuition.

Guess and intuition differ in their conscious status in another way, in that for guess, participants are not conscious that they have any relevant knowledge at all, but for intuition they are conscious of having relevant knowledge. That is, the knowledge expressed by the judgment that an item is grammatical is conscious in the case of intuition but not in the case of guess [33]. Using guesses rather than responses with any confidence (e.g., intuition) as an indicator of unconscious judgment knowledge goes back to the earliest studies of unconscious perception during the late 1800s and early 1900s (e.g., [35], [36]). More recently the distinction between guess and non-guess was made explicit as a distinct strategy for measuring the conscious status of perceptual judgments by Cheesman and Merikle [37], [38]. Specifically, Cheesman and Merikle proposed that unconscious perceptual judgments process should be defined in terms of subjective threshold, which is the point at which observers claim to detect perceptual information at no better than chance levels. Conversely, Reber (e. g., [1]), who coined the term implicit learning argued that intuition is the product of an implicit learning experience. In other words, participants with implicit grammatical knowledge have a sense of what is right or wrong without the ability to tell the reasons for that mental state: The unconscious structural knowledge expresses itself as intuition and not as a feeling of purely guessing. In sum, for guess, the participants do not know that they know, thus, judgment knowledge remains unconscious. For intuition, the participant knows that they know, therefore, judgment knowledge is conscious.

There is evidence that guesses pick up a qualitatively different sort of knowledge than intuition responses [4], [39]–[41]. Scott and Dienes [4] found that transfer between different concrete domains in an artificial grammar learning paradigm only occurred when grammaticality judgments were attributed to guess rather than those were attributed to conscious decisions (e.g., intuition). Mealor and Dienes [40] found incubation effects in artificial grammar learning only for guess responses and not others (e.g., intuition). Further, Mealor and Dienes found that time constraints affected guess and intuition responses in quite different ways, showing that response deadlines only interfered with the quality of intuition responses but not with guess responses ([39]; see also [41]). Thus, empirically and theoretically, it appears that guess and intuition responses have different functional properties.

These differences between guess and intuition responses were found in AGL tasks (see also [42]). The structures used in the artificial grammar learning paradigm concern only form-form mappings [10]. On the other hand, regularities underlying natural language usually concern form-meaning mappings as meanings lie at the core of language [10]. People tend to express their unconscious knowledge of natural language regularities with feelings of intuition. Thus, another major goal of the current study was to explore whether transfer of unconscious structural knowledge between domains is best facilitated when judgment knowledge is conscious or unconscious in a form-meaning connections task.

In summary, our primary goal in this article was to test whether people could implicitly transfer knowledge of form-meaning connections between metaphorically related domains bidirectionally. In addition, we sought to further explore whether transfer of unconscious knowledge across different domains is best facilitated when judgment knowledge is conscious or unconscious.

## Method

### Design

The design was a mixed 2×2 design, with a between participants factor of training domain (concrete versus abstract) and a within participants factor of generalization domain (same versus different).

### Participants

Thirty two volunteers (21 females) with an average age of 21.72 years (*SD* = 2.91) from the university community participated in the experiment in exchange for credits or 30 RMB. They were randomly allocated to one of the two training conditions, with 16 in the concrete training condition, 16 in the abstract training condition. The protocol used in this experiment was approved by the Ethics Committee of the Shanghai Psychological Society. Written informed consent was obtained from all participants in the experiments.

### Materials

Four Chinese characters 乜, 乇, 疋 and 夬 with frequencies lower than 1/1000,000 [43] were taken as determiners in their reduplicated forms (e.g.,乜乜的) to modify nouns. None of our participants knew the actual meaning of these characters. In addition, 80 nouns were selected. Among them, 40 nouns were clear cases of big or small objects (e.g. plaza or plate), 20 nouns of each class. Half of each class was randomly assigned to the training or testing phase. The remaining 40 nouns were clear cases of powerful or powerless social roles (e.g. king versus soldier), 20 nouns of each class. Half of each class was randomly assigned to the training or testing phase.

An independent sample of participants (N = 14) rated nouns on 7-point bipolar scales (see table 1) (for example, for valence, from 1 “very unpleasant” to 7 “very pleasant”). Big objects were perceived as bigger (*M* = 5.42, *SD* = .48) than small objects (*M* = 2.73, *SD* = .36), *t* (38) = 20.05, *P*<.01. We also computed the absolute difference between the size rating of each word and the neutral midpoint 4. The differences were significant, *t* (19) = 13.12, *P*<.01 for big object nouns, *t* (19) = 15.98, *P*<.01 for small object nouns respectively. Familiarity, valence and height of objects specified by object nouns were controlled (all *ps*>.1). A non-significant result in itself cannot be used to assert the null hypothesis. Thus, we analyzed the results further with a Bayes Factor [44], [45]. A Bayes Factor compares two theories, in this case the null hypothesis (that there are no differences in ratings of familiarity, valence and height between nouns denoted big objects and nouns denoted small objects) and the alternative hypothesis that there exist differences. A Bayes Factor is a number between 0 and infinity, where values greater than 3 indicate strong evidence for the alternative hypothesis, numbers less than 1/3 indicate strong evidence for the null, and numbers between 1/3 and 3 indicate data insensitivity (see [45]). Since the difference in size ratings between nouns denoting big objects and those denoting small objects was 2.69, we modeled an expectation for difference with a half-normal with a mode of zero and a standard deviation of 2.69 (following the recommendations of [45], Appendix). The Bayes Factors in favour of the existence of a difference over the null hypothesis of no difference were .01, .09 for ratings of familiarity and valence respectively, strongly supporting the null hypothesis. For ratings of height, the Bayes Factor was 0.83, indicating no sensitivity in the data for picking up whether or not there was difference. Maybe people confounded the concepts of height and size [22], or maybe they did not, the data do not say.

**Table 1:** Stimulus Characteristics (*M ± SD*).

	training				test			
	Concrete training domain (objects)		Abstract training domain (social roles)		objects		Social roles	
	Big	Small	Powerful	Powerless	Big	Small	Powerful	Powerless
valence	4.55±.52	4.55±.30	4.49±.51	4.29±.29	4.31±.64	4.46±.29	4.37±.39	4.33±.31
familiarity	6.18±.09	6.18±.08	6.14±.63	6.19±.15	6.19±.08	6.18±.09	6.18±.09	6.11±.28
height	5.07±.12	4.68±.81			5.13±1.11	4.50±.81		
size	5.59±.58	2.77±.31			5.24±.30	2.68±.41		
power			6.06±.49	2.77±.33			5.83±.36	2.95±.34

Powerful social roles were perceived as more powerful (*M* = 6.03, *SD* = .47) than powerless ones (*M* = 2.84, *SD* = .40), *t* (38) = 23.21, *P*<.01. We also computed the absolute difference between the power rating of each word and the neutral midpoint 4. The differences were significant, *t* (19) = 19.34, *P*<.01 for powerful social roles, and *t* (19) = 13.07, *P*–<.01 for powerless ones respectively. Familiarity and valence were controlled (all *ps*>0.1). Since the difference between power ratings between nouns specified powerful and powerless social roles was 3.20, we modeled an expectation for difference with a half-normal with a mode of zero and a standard deviation of 3.20. The Bayes Factor for ratings of familiarity and valence were .01 and .15 respectively, strongly supporting the null hypothesis in each case.

For the concrete training domain, there were two critical rules guiding the determiners before nouns: the distance rule and the size rule. The distance rule was explicitly introduced to participants, specifying whether the objects denoted by the noun phrases were relatively near to or far from the subject. What they were not told was the size rule, which specified whether the objects denoted by the noun phrases were big (e.g., plaza) or small (e.g., plate) in space. In one version of the materials, “乜” was designed to modify big and near objects, “乇” to small and near objects, “疋” to big and far objects, and “夬” to small and far objects. For example, “乜乜的广场”means “the square is near to the subject”, while “疋疋的广场”means “the square is far from the subject”. A second version of the materials was constructed, in which assignments of the determiners were reversed with respect to size, so that “乜” and “疋” modified small objects and “乇” and “夬” modified big objects. Half of the participants were tested on each version. Ten nouns of each size category (big vs. small) assigned for training occurred with both possible determiners (e.g., “乜乜的广场” and “疋疋的广场”), resulting in 40 training items in all (see Appendix S1).

For the abstract training domain, materials were constructed in a similar way with the exception that the hidden rule was about power rather than size and so nouns denoted powerful and powerless social roles (e.g., ruler or soldier) rather than objects. There were 40 training items in all (see Appendix S2).

The materials for the test phase consisted of 40 noun phrases without a determiner (e.g., _轮船“steamship”, _国王“king”; see Appendix S3). None of the nouns used for testing had been presented during the training phase. Twenty nouns denoting big or small objects were referred to as same domain generalization for the concrete training domain but different domain generalization for the abstract training domain. Another 20 nouns denoting powerful or powerless social roles were referred to as same domain generalization for the abstract training condition but different domain generalization for the concrete training condition. Each noun phrase was tested in both near and far situations. Thus, there were 80 testing items in all.

### Procedure

#### Vocabulary pretraining

Initially, participants had to learn four new characters (乜, 乇, 疋 and 夬) and were told that they were used as determiners to portray the relative distance of an object from them. Participants were presented with each character and asked to give its pronunciation and meaning (near or far) until they could go through all four characters without error.

#### Training

Each noun phrase was presented one at a time on the computer screen. Participants were instructed to (a) read the noun phrase aloud and give the meaning of the phrase, (b) form a mental image of the phrase while reading it three times and (c) press the corresponding key(d/k) to indicate whether it means “near” or “far”. For example, a trial in the concrete training domain could be “乜乜的广场”, where the participant (a) reads “mó mó de guǎng chǎng”, (b) indicates the meaning as “近的广场”(the near plaza), (c) imagines the phrase as the plaza being near while reading aloud the phrase three times, and finally (d) presses “d”. Accuracy feedback was provided. The presentation order of the training set was randomized.

#### Testing

Immediately after the training phase, the participants were tested on the size rule by a phrase completion task. For each trial, the computer displayed one phrase without a determiner (e.g., _轮船 “_steamship”) and two options below the phrase. The two options indicate the same distance but different size (e.g., both “乜乜的” and “乇乇的” indicated “near”, but “乜乜的” indicated “big” and “乇乇的” indicated “small”). For each trial, the participants were asked: (1) to choose one of the alternatives; (2) to rate their confidence in their choice on a scale from 50% to 100% (where 50% = 50% chance of being right or wrong, may as well flipped a coin; 100% = complete certainty) and (3) to indicate what they believed to be the basis for their decision (guess, intuition, memory or rules).

The trials of each generalization test were presented in the same order for half of the participants. The trial order was reversed for the other half. Test order (literal generalization first vs. metaphorical generalization first) was counterbalanced across participants.

## Results

### Proportion of correct responses

The proportion of correct response was calculated by 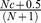 (*N_c_* being the number of correct responses; and *N* the total number of responses; see Bayesian Methods for explanation of the correction).

The overall percentage of correct response was 61% (*SD* = 12%), significantly above chance (50%), *t* (31) = 4.99, *p*<.001, *d* = .88, indicating that learning took place. Table 2 shows the means and *SD*s and confidence intervals for each experimental condition, resulting from orthogonal combination of training domain (concrete vs. abstract) and generalization domain (same vs. different); as can be seen, performance of each condition was above a chance baseline of .5, *ps*<.05, as the confidence intervals all exclude baseline.

**Table 2:** Mean accuracy of each condition.

Training domain	Concrete		Abstract	
Generalization domain	Same (concrete)	Different (abstract)	Same (abstract)	Different (concrete)
	0.62 (0.15) [0.54, 0.70]	0.65(0.17) [0.56, 0.74]	0.57(0.10) [0.52, 0.63]	0.59(.10) [0.54, 0.64]

Note: means with SDs in parentheses and 95% confidence interval in square brackets.

A 2 (training domain: concrete vs. abstract)×2 (generalization domain: same vs. different) general linear model (GLM) with repeated measures on the second factor revealed no significant main effect of group, *F* (1, 30) = 1.66, *p*>.05, *η^2^* = .05, of generalization domain, *F* (1, 30) = 1.57, *p*>.05, *η^2^* = .05, and no significant interaction between the two factors, *F* (1, 30) = .15, *p*>.05, *η^2^* = .005.

### Judgment knowledge

According to the guessing criterion [37], participants may think that they are guessing when in fact they perform above chance. The mean classification performance of each condition when participants gave a confidence rating of 50% is shown in Table 3. Comparisons between accuracy when participants gave a confidence rating of 50% and chance level in each experimental condition revealed no significant differences: *t* (15) = −1.37, *p*>.05, *d* = .34 for condition of concrete-to-concrete; *t* (15) = .12, *p*>.05, *d* = .03 for condition of concrete-to-abstract, *t* (15) = .88, *p*>.05, *d* = .23 for condition of abstract-to-abstract and *t* (15) = −0.53, *p*>.05, *d* = .14 for condition of abstract-to-concrete. While the results being non-significant appear not to satisfy the guessing criterion of unconscious knowledge, a non-significant result in itself cannot be used to assert the null hypothesis. Thus, we analyzed the result further with a Bayes Factor. In this case the null hypothesis (that all knowledge is conscious) and the alternative hypothesis that there exists some unconscious knowledge. To interpret a null result one needs to know what size effects could be expected if they existed. Chen et al [9] tested literal generalization in a similar paradigm (but for learning animacy rather than size) and found overall accuracy for responses based on unconscious structural knowledge for generalization items was 55% (in experiment 1). Thus, we modeled an expectation for unconscious knowledge with a half-normal with a mode of zero and a standard deviation of 5% (following the recommendations of [45], Appendix). For condition of concrete-to-concrete, the mean difference between the experimental and chance level in percentage correct for knowledge when participants gave a confidence rating of 50% was −6%, with a standard error of difference of 5%. The Bayes Factor in favour of the existence of unconscious knowledge over the null hypothesis of no unconscious knowledge was .36, above1/3, indicating limited sensitivity in the data for picking up whether or not there was unconscious knowledge (for other applications of Bayes to implicit learning, see [5], [40], [46]). The Bayes Factors in favour of the existence of unconscious knowledge over the null hypothesis of no unconscious knowledge for the other three conditions were .70 (concrete-to-abstract), 1.13 (abstract-to-abstract) and .46 (abstract-to-concrete), all above 1/3, suggesting no sensitivity in the data for picking up whether or not there was unconscious knowledge.

**Table 3:** Mean Accuracy When Participants Were Guessing (50% Confidence Rating) or Confident (51–100% Confidence Rating) and the Number of Participants Who Were Included in the Analysis.

Training domain	Generalization domain	Confidence degree	Response Proportion (%)	N	Response Accuracy
Concrete	Same (concrete-to-concrete)	Guess	0.17 (0.18)	16	0.44 (0.18)
		Confident to any degree	0.83 (0.18)	16	0.63 (0.17)
	Different (concrete-to-abstract)	Guess	0.18 (0.17)	15	0.51 (0.16)
		Confident to any degree	0.82 (0.17)	16	0.66 (0.18)
Abstract	Same (abstract-to-abstract)	Guess	0.21 (0.17)	14	0.53 (0.13)
		Confident to any degree	0.79 (0.17)	16	0.57 (0.13)
	Different (abstract-to-concrete)	Guess	0.27 (0.21)	14	0.48 (0.15)
		Confident to any degree	0.73 (0.21)	16	0.63 (0.13)

According to the zero-correlation criterion, knowledge is unconscious when people cannot distinguish states of guessing from states of knowing, as shown by no relation between accuracy and “guess” versus “some confidence” responses. In terms of showing unconscious knowledge, it is specifically the distinction between completely guessing and having any confidence that is important [47], so we divided confidence between 50% (pure guessing) and any other value (51–100%). Accuracy for such guess and confident responses is shown in Table 3. For condition of concrete-to-concrete, the difference in accuracy (the accuracy-confidence “slope”) was 20% (*SD* = 26%), was significant, *t* (15) = 3.03, *p*<.05, *d* = .76. For condition of concrete-to-abstract, the difference in accuracy was −16% (*SD* = 20%), was significant, *t* (14) = 3.10, *p*<.05, *d* = .80. For condition of abstract-to-concrete, the difference in accuracy was 16% (*SD* = 17%), was significant, *t* (13) = 3.50, *p*<.05, *d* = .94. These results indicated the existence of conscious judgment knowledge in the above three conditions. For the condition of abstract-to-abstract, the difference in accuracy was 3% (*SD* = 18%), non-significant, *t* (13) = .68, *p*>.05, *d* = .18. Since the non-significant result in itself cannot be used to assert the null hypothesis, we further analyzed the null results with a Bayes Factor. It can be shown that the maximum slope that can be obtained depends on the proportion of confident responses, pc; specifically if the overall accuracy (ignoring confidence) is X% above baseline, then the maximum slope possible is X/pc (see Bayesian Methods). For the current data, X = 12%, *pc* = .83, thus maximum slope = 14%. With this assumption the Bayes Factor was .80. That is, the data are insensitive, and nothing can be concluded about whether or not there was conscious knowledge for the condition of abstract-to-abstract, as measured by the zero correlation-criterion.

In short, people acquired conscious judgment knowledge, as measured by the zero-correlation criterion, in at least most of the conditions. Further, participants gave higher confidence ratings for intuition than guess attributions, *t*(30) = 10.57, *p*<.001, which is as expected as intuition was defined as being different from guess by virtue of the participants having confidence in their answer [33].

One problem with the guessing criterion is that people can be inconsistent in the subjective judgments that they give. Thus, when people give a source attribution of “guessing” they did not always give a confidence rating of 50% as they should by the definition of the terms [48]. However, such variability in confidence ratings may amount to either subjects belief that they should sometimes use different confidence ratings, or else to random variations in confidence with no basis in variations in actual metacognitive ability. If so, for guess attributions there should be no relation between confidence and accuracy. Indeed, for guess attributions, the difference in average confidence for correct and incorrect answers was not significant, *t* (30) = 1.29, *p*>.05, appearing to satisfy the zero correlation criterion of unconscious judgment knowledge. The maximum value this difference can be can be shown to be Y/pct, where Y is the amount by which average confidence is above pure guessing, and pct is the proportion of correct responses. For guesses, Y = 5.9% and pct = 0.61, so the maximum difference that could be obtained is 9.7%. Thus we modeled the hypothesis of a difference as a uniform between 0 and 9.7%. The Bayes Factor (using the online calculator for the website for Dienes, 2008) was 0.1, less than 1/3, indicating strong evidence for the null hypothesis, suggesting that there is no conscious judgment knowledge as measured by the zero correlation-criterion. For intuition responses, the difference of the average confidence for correct and incorrect answers was significant, *t*(29) = 2.24, *p*<.05, suggesting that participants did possess conscious judgment knowledge. These results support the assumption that guess responses involve unconscious judgment knowledge and intuition responses involve conscious judgment knowledge [33], even allowing for the natural inconsistency in participants' responses.

### Structural knowledge

**Figure 1:**
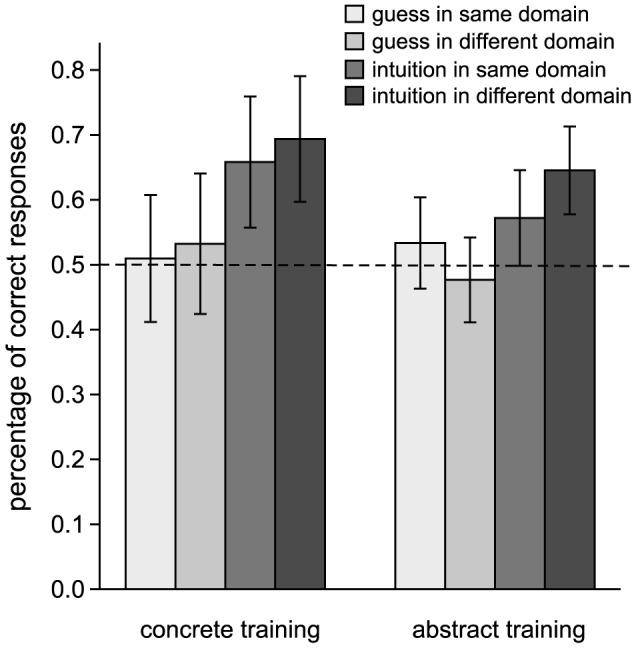
Percentage of correct responses by guess and intuition attributions for each condition. Error bars indicate 95% confidence interval.

Guess and intuition attributions indicate unconscious structural knowledge (implicit attributions), while memory and rule attributions indicate conscious structural knowledge (explicit attributions) [33]. The response proportions of each attribution are shown in Table 4. Since less than 5% of attributions reflected conscious knowledge, we did not include explicit attributions in the following analyses. The proportion of correct responses for guess and intuition attributions of each condition is shown in Table 5 and figure 1.

**Table 4:** Response proportions of each attribution for each condition (*M ± SD*).

Training domain	Generalization domain	Implicit attributions		Explicit attributions	
		guess	Intuition	memory	Rule
Concrete	same	0.300±0.314	0.697±0.314	0.003±0.009	0.000±0.000
	different	0.275±0.306	0.718±0.308	0.005±0.014	0.002±±0.006
Abstract	same	0.247±0.179	0.745±0.179	0.009±0.018	0.000±0.000
	different	0.317±0.187	0.672±0.191	0.011±0.027	0.000±0.000

**Table 5:** Percentage of correct responses attributed to guess and intuition for each condition.

Training domain	Generalization domain	Guess	Intuition
Concrete	same domain	0.51±0.18	0.66±0.18
	different domain	0.53±0.19	0.69±0.17
Abstract	same domain	0.53±0.13	0.57±0.14
	different domain	0.48±0.12	0.65±0.13

When guess and intuition were combined together for implicit attributions, the overall percentage of correct percentage was 61% (*SD* = 12%), significantly above chance (50%), *t* (31) = 5.07, *p*<.001, *d* = .90, indicating people acquired unconscious structural knowledge.

To test our major hypotheses about the bidirectional transfer effect, we calculated the accuracy of implicit attributions of each experimental condition, resulting from orthogonal combination of training domain and generalization domain. As predicted, the accuracy of implicit attributions in each condition was significantly above chance (*t* (15) = 3.25, *p*<.01, *d* = .81 for concrete-to-concrete; *t* (15) = 3.57, *p*<.01, *d* = .89 for concrete-to-abstract; *t* (15) = 3.74, *p*<.01, *d* = .94 for abstract-to-concrete; *t* (15) = 2.89, *p*<.05, *d* = .72 for abstract-to-abstract), suggesting that people can unconsciously transfer knowledge of form-meaning connections between concrete and abstract domains bidirectionally.

To explore whether transfer of form-meaning connections between metaphorically related domains is best facilitated when judgment knowledge is conscious (intuition) or unconscious (guess), we conducted a 2 (training domain: concrete vs. abstract)×2 (generalization domain: same vs. different)×2 (structural knowledge: guess vs. intuition) general linear model (GLM) with repeated measures on the last two factors. The analysis yielded a significant main effect of structural knowledge, *F* (1, 25) = 26.96, *p*<.01, *η^2^* = .52. Performance based on intuition responses was significantly better than that based on guess. Further analysis showed that performance based on intuition (*M* = .65, *SD* = .14) was significantly better than chance, *t* (26) = 5.62, *p*>.01, *d* = 1.08 while that based on guess (*M* = .52, *SD* = .13) was not significantly better than chance, *t* (26) = .77, *p*>.05, *d* = .15. We also analyzed the null results of performance based on guess responses with a Bayes Factor. We modeled an expectation for performance attributed to guess with a half-normal with a mode of zero and a standard deviation of 5% to calculate the Bayes Factor according to performance based on unconscious structural knowledge of Chen et al [9] as mentioned before. The Bayes Factor was .87, indicating limited sensitivity in the data for picking up whether or not performance attributed to guess is different from chance. No other significant main effects or interactions were found. Confirming that the advantage of intuition over guess responses applied even to the transfer case (different generalization) a t-test revealed *t* (26) = 4.58, *p*<.01, *d* = .82. Further analysis showed that performance based on intuition (*M* = .68, *SD* = .16) was significantly better than chance, *t* (26) = 5.93, *p*>.01, *d* = 1.14 while that based on guess (*M* = .51, *SD* = .16) was not significantly better than chance, *t* (26) = .30, *p*>.05, *d* = .06. We also analyzed the null results of performance based on guess responses with a Bayes Factor with the same logic above. The Bayes Factor was .66, indicating limited sensitivity in the data for picking up whether or not performance attributed to guess is different from chance in transfer. These results suggest that transfer of form-meaning connections between metaphorically related domains was best facilitated by intuition rather than guess, showing a different pattern from that found in an AGL task [4].

## Discussion

This study investigated the bidirectional transfer between superficially dissimilar but metaphorically related domains with an implicit learning paradigm for investigating form-meaning connections. The results showed that people can transfer acquired knowledge of form-meaning connections from a concrete domain “size” to an abstract one “power” (in the concrete training condition) and vice versa (in the abstract training condition). Importantly, transfer occurred when the structural knowledge underlying the judgment is unconscious. Furthermore, the current study found that transfer between metaphorically related domains is best facilitated by conscious rather than unconscious judgment knowledge (while structural knowledge remains unconscious).

Previous studies [9], [11] have shown similar implicit learning effects for the animacy distinction but not for a distinction based on relative size. By contrast, the present study demonstrated that people learned a size distinction, in line with our previous study showing that people learned a height distinction [19]. Leung and Williams [11] proposed two possible explanations for why the relative size distinction is less amenable to implicit learning in this paradigm: a conceptual availability account and a linguistic relevance account. The conceptual availability account is built on the argument that there is an evolutionary advantage in detecting animacy, making the concept chronically conceptually available [49] (see also [50]). Accordingly, Leung and Williams argued that the rapid automatic activation of animacy might have allowed implicitly associating form and meaning [11]. However, relative size is not an intrinsic object property and has to be computed, introducing processes taking time and thus reducing learning of form-meaning connections [11]. According to this account, a property of evolutionary value is more amenable to implicit learning. In the current case, the regularity is about the natural distinction of size, which is relevant for surviving. As Freedman noted, “throughout nature the rule is the bigger, the more dangerous”(p. 29) [51]. The ratings of size by an independent sample (used in the nouns standardization phase) indicated that the big/small distinction of the objects is salient (see details in Materials). Therefore, an automatic distinction was plausibly automatically made by subjects between big/small objects. In contrast, the size regularities used in previous studies - the relative size of two objects [11] or size of an animal relative to a dog [9] - are either not intrinsic features of an object or not ones likely to be evolutionarily selected for automatic encoding. For example, a pig was large and a monkey was small in [9]. But people may not perceive a pig as large or a monkey as small automatically. Thus, the arbitrariness of the distinction based on size might reduce the likelihood of establishing a form-meaning connection.

An alternative explanation proposed by Leung & Williams [11]was that linguistic relevance might be a critical factor in implicit learning (also see in [9]). According to this account, people can learn form-meaning connections based on animacy because animacy is the kind of information existing in the participants' prior grammatical knowledge, while relative size is not. This account also makes sense in our case. Although in Chinese, size is not actually grammaticized, it does operate as a constraint on the use of some classifiers. For example, “粒” usually modifies small objects (e.g., 一粒米 “a grain of rice”), while “艘” usually modifies big objects (e.g., 一艘船 “a ship”). However, there is no Chinese classifier sensitive to relative size nor to size relative to a dog. In short, conceptual availability and linguistic relevance cannot yet be distinguished as an explanation for what is implicitly learned in the form meaning paradigm, and this would be an interesting area for future research to explore. Whether conceptual availability or linguistic relevance turns out to contribute to learning, each is an example of the relevance of prior knowledge and expectations in determining what is implicitly learned [52].

The current results replicated prior findings that people can unconsciously learn form-meaning connections [9]–[11] as well as transfer knowledge of form-meaning connections from a concrete domain to an abstract one in a metaphor-consistent way [19]. More importantly, this study extended Li et al's work by showing that unconscious transfer of knowledge from an abstract domain to a more concrete one also occurred. These results demonstrated that implicitly acquired knowledge appears to flexibly transfer between superficially different domains. These findings challenge the assumption that metaphor has cognitive asymmetry or directionality (e.g., [22]). Instead, the present results are in line with the assumption that metaphorical influences are bidirectional. This could be explained from the embodiment perspective which posits that metaphors reflect conceptual knowledge grounded in perceptual experience (e.g. [53]), and conceptual processing involves perceptual simulation which may activate elements of an underlying knowledge structure [31]. Accordingly, in the current study, information about size might have activated the concept of power in the concrete learning condition, while information about power might also have activated that of size in the abstract learning condition. It seems reasonable to believe that such co-activation of metaphorically related domains cause the bidirectional transfer effect in our study. However, a question that remains open is whether perceptual simulation or semantic activation induces the bidirectional effect in the present study. This issue is worthy of further investigation, but it lies beyond the scope of the present study.

Moreover, the current results shed light on the difference between conscious and unconscious judgment knowledge when structural knowledge is unconscious. Dienes argued that conscious and unconscious structural knowledge are fundamentally different in contents and learning mechanisms [44], [54]. Such an account leaves open the possibility that when structural knowledge is unconscious, the difference between conscious and unconscious judgment knowledge is graded and flexible, with an easy transition between the two (e.g., [48], [55]). Nonetheless, recent evidence suggests that conscious and unconscious judgment knowledge may have different functional properties as mentioned before [4], [39]–[41]. Our data is consistent with Mealor and Dienes [39]–[41] in terms of finding a dissociation between guess and intuition. However, with regard to transfer, the present study is inconsistent with that of Scott and Dienes [4]. In particular, Scott and Dienes' study [4] found transfer between different concrete domains only for random (guess) responses rather than those attributed to conscious decisions (e.g., intuition). With a form-meaning connections task, the present study found that transfer between different domains was best facilitated by intuition rather than guess.

Some difference between the current study and [4] may help to explain the inconsistent results: First, the underlying regularities in [4] were generated from a novel finite state grammar. In contrast in our study, people have pervasive experience of the metaphorical mappings between size and power underlying the stimuli, either in language or in thinking [22], [23]. As Dienes and Scott argued that with further domain experience, people may come to know that they have relevant knowledge, and thus have conscious judgment knowledge even while structural knowledge remains unconscious as in natural language [33].

Second, in [4], a complex set of rules was employed to generate the stimuli, such as “the string starts with letter V or M”, “The T can be immediately repeated after an V.”, etc. However, the regularity in our study can be simply described as “乇 and 夬 occur with big objects or powerful social roles, 疋 and 乜 with small objects or powerless social roles”, which is simpler than the rules in previous work [4]. Thus, the limited amount of material to be learnt in our study may allow more awareness of having knowledge. Indeed, Williams found that 7 out of 37 participants (experiment1) and 7 out of 24 participants (experiment2) expressed awareness of the underlying rule in this paradigm [13].

Third, in [4], participants transferred knowledge from one concrete perceptual domain to another (e.g., letter–letter; note–letter; note-symbol). In contrast, people had to transfer knowledge from a concrete perceptual domain (size) to an abstract one (power) or vice versa. Analysis of the results of [4] revealed that the additional accuracy shown in grammaticality judgments attributed to random selection may arise, at least in part, from a greater sensitivity to chunk novelty. Scott and Dienes argued that guess responses, perhaps based upon chunk novelty, might be obscured by the attempt to access conscious feelings of intuition [4]. This is likely to be the reason why knowledge was expressed predominantly in the random attributions. Combined with previous study [4] and the current results, it is possible that guess responses may better express concrete structural knowledge and have difficulty with more abstract relations, such as the metaphorical mappings between concrete and abstract domains in our case. But conscious “fringe feelings” [56] like intuition may express unconscious structural knowledge of both perceptual and more abstract relations. These possibilities are worth of further investigation

In conclusion, the current results provided new evidence for the flexibility of unconscious knowledge [1], [2] as well as the bidirectional perspective of metaphors [31]. Moreover, our results also revealed a distinction between guess and intuition in showing that transfer between metaphorically related domains in form-meaning connections paradigm occurred more readily when judgment knowledge was conscious (intuition) rather than unconscious (guess), which is different from what was found in [4]. These results suggest that conscious and unconscious judgment knowledge may have different functional properties, depending on the domain that people are trained on.

## Bayesian Methods

### Correction for proportions

The correction of adding 0.5 to the numerator and 1 to the denominator of the proportion corresponds to a Bayesian prior of chance performance worth just one observation (a “unit information prior” [57]).When there is low N even vague prior information increases accuracy of estimates (e.g., [44]). In particular, adding one observation in total where it is 0.5 correct corresponds to being 95% confident that the proportion is between 5% and 95%, and most likely near the middle of the range. Even though very vague, the prior nonetheless reflects our knowledge that in this sort of paradigm performance is somewhere near 50–60%. Without the correction, if the data consisted of one observation, the estimated proportion is either 0% or 100%. We know either value is false. With the prior added, the proportion would be estimated as 25% or 75%, either value being more accurate by virtue of the added prior. As N increases, the prior becomes swamped by the data.

### Finding the maximum possible for the accuracy confidence slope

A Bayes factor requires a specification of the range of effect sizes that can be plausibly expected. It can be shown that the maximum slope that can be obtained depends on the proportion of confident responses, pc; specifically if the overall accuracy (ignoring confidence) is X% above baseline, then the maximum slope possible is X/pc. X is a weighted average of the performance above baseline when guessing (G) and when confident (C), with the weights being the proportions of each type of response. That is, X = (1−pc)*G+pc*C. By definition, our measure of confidence accuracy relation, the slope, is C–G. This will be maximum when all guessing responses are at baseline, i.e. when G = 0. In this case, slope = C–G = C. Also in this case, X = pc*C, with the G term dropping out. Rearranging, C = X/pc. Thus, since maximum slope = C in this case, maximum slope = X/pc. Thus, the theory that there exists some conscious knowledge can be represented as a uniform between 0 and X/pc (see also [19] and [58] for previous uses of this method for evaluating the zero correlation criterion). That is, conscious knowledge, if it exists, is assumed to be possibly any value from infinitesimally small to the maximum allowed.

### Finding the maximum possible for the Chan difference score

In the previous section the relation between confidence and accuracy was expressed as a difference in accuracy between guess and confident responses. Conditionalizing accuracy on pure guess versus some confidence is the best way of exploring the zero correlation criterion in most circumstances [47]. However, when people are already claiming they are guessing, but still give variations in fine grained confidence, it is possible to test for difference in average confidence between correct and incorrect answers (the Chan difference score). To interpret a non-significant Chan difference score, a Bayes factor is needed, and thus it is useful to know what the plausible range of the scale is. If the overall confidence (ignoring accuracy) is Y% above baseline, then the maximum Chan difference score possible is Y/pct, where pct is the proportion of correct responses, as shown by an exactly analogous argument as in the previous section: Y is a weighted average of the confidence above baseline when not correct (NC) and when correct (C), with the weights being the proportions of each type of response (pct = proportion correct). That is, Y = (1−pct)*NC+pct*C. By definition, our measure of confidence accuracy relation, the Chan difference score, is C – NC. This will be maximum when all not correct responses are at baseline, i.e. when NC = 0. In this case, Chan difference score = C – NC = C. Also in this case, Y = pct*C, with the NC term dropping out. Rearranging, C = Y/pct. Thus, since maximum Chan difference score = C in this case, maximum Chan difference score = Y/pct.

## Supporting Information

Appendix S1 **Training items for concrete training domain.**
(DOC)

Appendix S2 **Training items for abstract training domain.**
(DOC)

Appendix S3 **Test items.**
(DOC)
