# Supplementary figures and images for: Bidirectional Transfer between Metaphorical Related Domains in Implicit Learning of Form-Meaning Connections
Source: PLoS One. 2013 Jul 3;8(7):e68100. doi: 10.1371/journal.pone.0068100 (PMC3701079; doi:10.1371/journal.pone.0068100)

Appendix S1. Training items for concrete training domain.


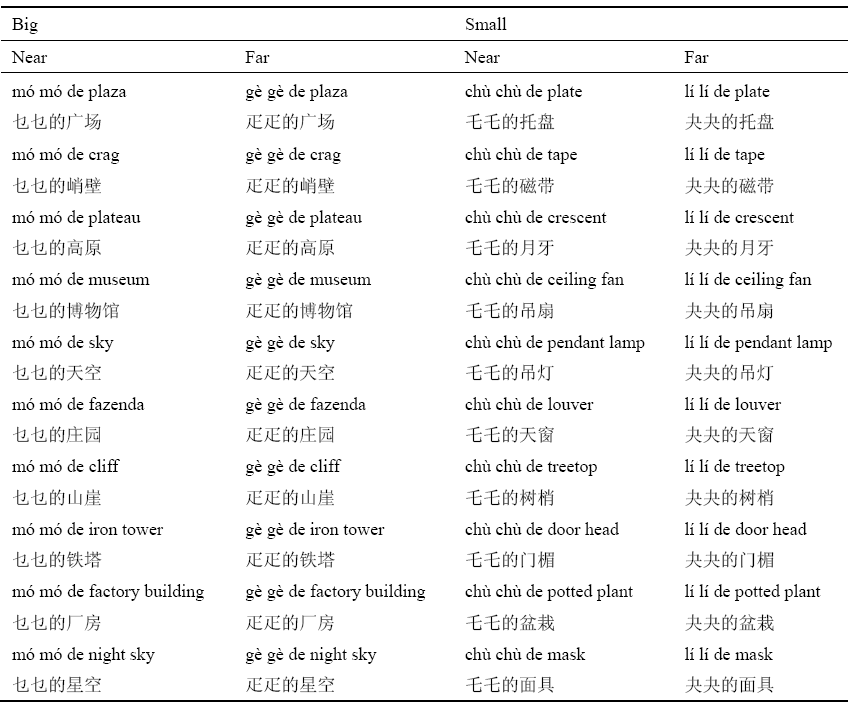

Supplement: Appendix S1 — Training items for concrete training domain. (DOC) [file pone.0068100.s001.doc]

Appendix S2. Training items for abstract training domain.


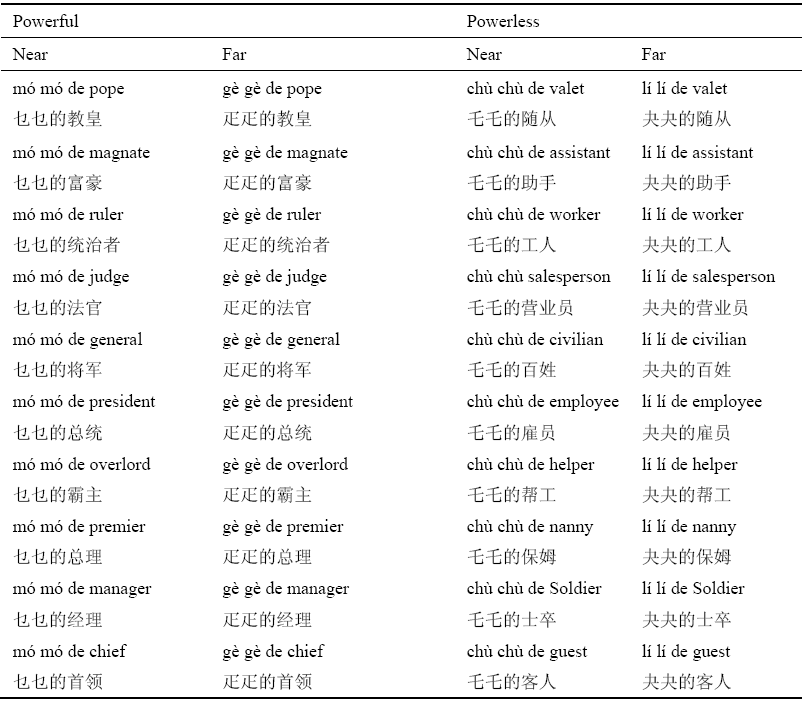

Supplement: Appendix S2 — Training items for abstract training domain. (DOC) [file pone.0068100.s002.doc]

Appendix S3. Test items.


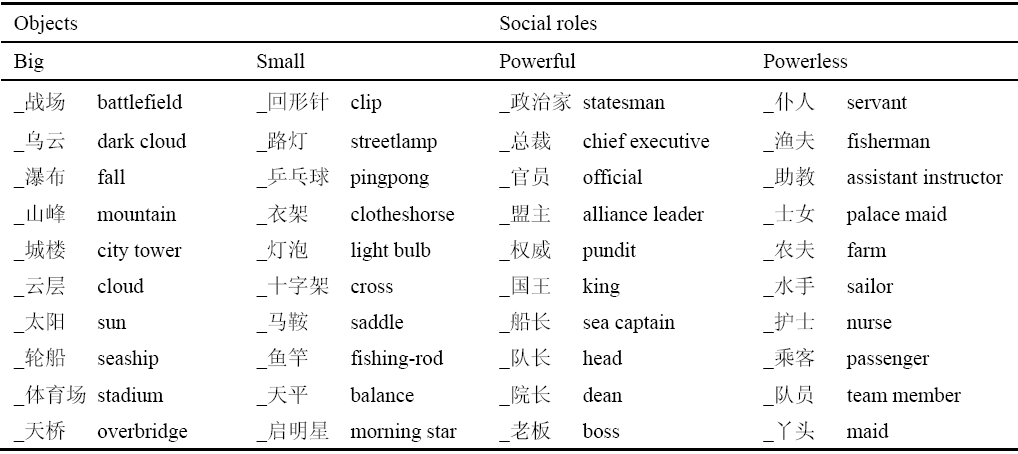

Supplement: Appendix S3 — Test items. (DOC) [file pone.0068100.s003.doc]
